# Tracheid cell-wall structures and locations of (1 → 4)-β-d-galactans and (1 → 3)-β-d-glucans in compression woods of radiata pine (*Pinus radiata* D. Don)

**DOI:** 10.1186/s12870-016-0884-3

**Published:** 2016-09-07

**Authors:** Miao Zhang, Ramesh R. Chavan, Bronwen G. Smith, Brian H. McArdle, Philip J. Harris

**Affiliations:** 1 School of Biological Sciences, The University of Auckland, Private Bag 92019, Auckland Mail Centre, Auckland, 1142 New Zealand; 2 School of Chemical Sciences, The University of Auckland, Private Bag 92019, Auckland Mail Centre, Auckland, 1142 New Zealand; 3 Department of Statistics, The University of Auckland, Private Bag 92019, Auckland Mail Centre, Auckland, 1142 New Zealand

**Keywords:** Immunomicroscopy, Monoclonal antibodies, Opposite wood (OW), Mild compression wood (MCW), Neutral monosaccharide compositions, Non-cellulosic polysaccharides, Plant cell walls, Reaction wood, Severe compression wood (SCW)

## Abstract

**Background:**

Compression wood (CW) forms on the underside of tilted stems of coniferous gymnosperms and opposite wood (OW) on the upperside. The tracheid walls of these wood types differ structurally and chemically. Although much is known about the most severe form of CW, severe CW (SCW), mild CWs (MCWs), also occur, but less is known about them. In this study, tracheid wall structures and compositions of two grades of MCWs (1 and 2) and SCW were investigated and compared with OW in slightly tilted radiata pine (*Pinus radiata*) stems.

**Results:**

The four wood types were identified by the distribution of lignin in their tracheid walls. Only the tracheid walls of OW and MCW1 had a S3 layer and this was thin in MCW1. The tracheid walls of only SCW had a S2 layer with helical cavities in the inner region (S2i). Using immunomicroscopy, (1 → 4)-β-D-galactans and (1 → 3)-β-D-glucans were detected in the tracheid walls of all CWs, but in only trace amounts in OW. The (1 → 4)-β-D-galactans were located in the outer region of the S2 layer, whereas the (1 → 3)-β-D-glucans were in the inner S2i region. The areas and intensities of labelling increased with CW severity. The antibody for (1 → 4)-β-D-galactans was also used to identify the locations and relative amounts of these galactans in whole stem cross sections based on the formation of an insoluble dye. Areas containing the four wood types were clearly differentiated depending on colour intensity. The neutral monosaccharide compositions of the non-cellulosic polysaccharides of these wood types were determined on small, well defined discs, and showed the proportion of galactose was higher for CWs and increased with severity.

**Conclusion:**

The presence of an S3 wall layer is a marker for very MCW and the presence of helical cavities in the S2 wall layer for SCW. The occurrence and proportions of (1 → 4)-β-D-galactans and (1 → 3)-β-D-glucans can be used as markers for CW and its severity. The proportions of galactose were consistent with the labelling results for (1 → 4)-β-D-galactans.

**Electronic supplementary material:**

The online version of this article (doi:10.1186/s12870-016-0884-3) contains supplementary material, which is available to authorized users.

## Background

Cell walls of the secondary xylem of woody plants are of considerable commercial importance. In addition to being the major component of solid wood, they are used to produce pulp for making paper and second generation liquid biofuels. When the growth of stems of such woody plants is displaced from the vertical, for example by wind or snow, a special type of secondary xylem is formed known as reaction wood, which restores normal, vertical growth [1, 2]. In coniferous gymnosperms (softwoods), this reaction wood is formed on the underside of tilted stems and is known as compression wood (CW) [2]. The cell walls in this wood contain more lignin and less cellulose than normal wood (NW) [3–5]. The wood formed geometrically opposite to CW, is known as opposite wood (OW) and the cell walls of this wood type are structurally and chemically similar to those of NW [4]. Lignin hinders the production of chemical pulps and biofuels, and consequently the presence of CW reduces their yields [6]. Additionally, the presence of CW affects the quality of solid wood. On drying, CW shrinks longitudinally more than NW, and when CW occurs with NW, the differential shrinkage causes warping and other distortions [7].

The wood of coniferous gymnosperms consists mostly of tracheids that have a dual role: they conduct water up the stem and they provide mechanical support. Tracheid cell walls are composed of a thin primary wall layer and a thick secondary wall. The individual tracheids adhere to one another by a thin middle lamella (ML), and this together with the two adjacent primary walls are often referred to as the compound middle lamella (CML) [8, 9]. Tracheid walls of CW differ both structurally and chemically from NWs and OWs [2, 4, 10, 11]. In tracheids of NW and OW, the secondary walls are composed of three layers, S1, S2 and S3, with the S2 layer the thickest and the S3 layer adjacent to the cell lumen [8, 9]. In CW, the tracheid walls are thicker, but lack the S3 layer. The S2 layer often has helical cavities on the inner side nearest the cell lumen. CW also differs from NWs and OWs in the presence of intercellular spaces at the corners of adjacent tracheids.

Chemically, the tracheid walls of NWs and OWs are composed of cellulose, lignin and the non-cellulosic polysaccharides heteromannans (*O*-acetyl-galactoglucomannans) and smaller proportions of heteroxylans [arabino(4-*O*-methylglucurono)xylans] [4, 12]. In addition to containing less cellulose and more lignin, the tracheid walls of CW contain less heteromannans, and heteroxylans. These walls also contain significant proportions of (1 → 4)-β-D-galactans (up to 10 %) and small proportions (~3 %) of (1 → 3)-β-D-glucans (callose or laricinan) [2, 4, 13]. The greater longitudinal shrinkage of CW has been correlated with these (1 → 4)-β-galactans [4, 14].

In addition, the distribution of lignin in tracheid walls of CW differs from that in NW and OW. Lignin is autofluorescent in ultraviolet and blue radiation and can be localized using fluorescence microscopy. Using this technique it has been found that lignin occurs at high concentrations in the ML and primary walls of tracheids in OW and NW. However, in CW, the lignin concentrations in the ML and primary walls are lower, but are higher in the outer region of the S2 layer (S2L) [15]. CW is also often darker in colour than NWs and OWs.

CW, as described above, is more accurately described as severe CW (SCW), but a continuum of wood types (grades) occur between this and NW or OW, with the intermediate types being referred to as mild CWs (MCWs) [11]. As many as five major grades of CWs (one severe and four mild) have been described in white spruce (*Picea glauca*) [16]. Different CW grades can most reliably be recognized based on the distribution of lignin in tracheid walls as determined by its autofluorescence using fluorescence microscopy [11]. However, there is currently no other good method available to accurately detect and classify CW on a larger scale, i.e. with the naked eye or low-power (stereo) microscope. Compared with SCW, there have been only a few studies of mild CWs and relatively little is known about the relationship between structural and chemical features in the walls of different grades of CW. However, one study correlated structural features with chemical analyses in radiata pine (*Pinus radiata*), but quite large samples of wood (2–3 g) were analysed, and within this, the structural features were so highly variable that only one MCW grade was identified [17].

Here, we describe a study in which saplings of radiata pine were grown tilted from the vertical to induce the formation of CW. A small tilt angle (~8–20°) to the vertical was used to try to maximize the formation of MCW rather than SCW. Four wood types, OW, SCW and two MCWs, were identified based on the distribution of lignin in their tracheid walls using fluorescence microscopy. Light and electron microscopy were used to compare the structures of the tracheid walls of the four wood types and immunomicroscopy with monoclonal antibodies was used to specifically locate (1 → 4)-β-galactans and (1 → 3)-β-glucans in relation to structural features in the walls. Immunolabelling with an enzyme labelled secondary antibody was used to examine the distribution of (1 → 4)-β-galactans in whole cross sections of stems. Pure, synthetic aniline blue fluorochrome that binds specifically to (1 → 3)-β-glucans [18] was also used with fluorescence microscopy to locate this polysaccharide in the tracheid walls. Additionally, the neutral monosaccharide compositions of the non-cellulosic cell-wall polysaccharides of the four wood types were determined using small discs (0.5 mm diameter) each containing only a single wood type as determined by fluorescence microscopy.

## Results

### Three grades of CW were identified by lignin distribution in tracheid walls

**Fig. 1:**
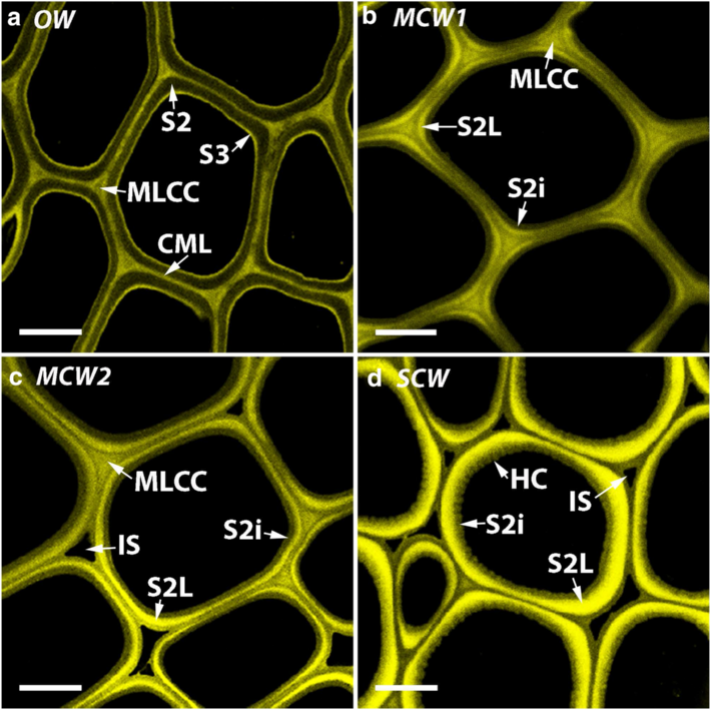
Fluorescence micrographs of transverse sections of OW and three CWs showing lignin autofluorescence. In OW tracheid walls (**a**) lignin autofluorescence is strongest in the compound middle lamella (CML), the middle lamella at the cell corners (MLCC), and in the S3 layer of the secondary walls. In MCW1 (**b**), there is moderate autofluorescence in the middle lamella at the cell corners, and some autofluorescence in the S2L region (S2L) at the cell corners. In MCW2 (**c**), autofluorescence of the S2L layer at the cell corners is more evident and this layer is also fluorescent around the cell. In SCW (**d**) there is a highly fluorescent S2L layer all around the tracheids and there is no fluorescence of the CML. Helical cavities (HC) are present on the inner region of the S2 layer (S2i). Intercellular spaces (IS) are present in MCW 2 and SCW. Sections were from Tree 1 and the micrographs obtained using a Leica confocal microscope. Scale bar: 10 μm

Three grades of CW were identified in transverse sections of stems of tilted saplings of radiata pine based on the distribution of lignin autofluorescence in their tracheid walls: MCW 1 and 2, and SCW (Table 1, Fig. 1). In OW tracheids, lignin autofluorescence was most evident in the CML and in the ML at cell corners (MLCC), and in the S3 layer of the secondary walls (Fig. 1a). In MCW1 tracheids, some autofluorescence was present in the S2L layer at the cell corners (Fig. 1b). However, in the MCW2 tracheids, this autofluorescence of the S2L layer at the cell corners was more evident and this layer was also present around the cells (Fig. 1c). In SCW tracheids, the intensity of the S2L layer fluorescence was greater and there was no fluorescence of the CML, either at the cell corners (MLCC) or around the cells (CML) (Fig. 1d). In addition, intercellular spaces were identified only between MCW2 and SCW tracheids, and the tracheids became increasingly circular in transverse section on going from OW to SCW.

**Table 1:** Characteristics of the three grades of compression woods (CW) and opposite wood (OW)

	Characters			
Wood types	Lignification of compound middle lamella at cell corners	Lignification of S2L at cell corners	Lignification of S2L around cells	Intercellular spaces
Opposite wood (OW)	+++	-	-	-
Mild compression wood 1 (MCW1)	++	+	-	-
Mild compression wood 2 (MCW2)	+	++	++	++
Severe compression wood (SCW)	-	+++	+++	+++

-, no lignin autofluorescence/intercellular spaces absent; +, weak lignin autofluorescence; ++, moderate lignin autofluorescence/moderately frequent intercellular spaces; +++, strong lignin autofluorescence/numerous intercellular spaces

When transverse sections of whole sections of the tilted saplings were examined in reflected light, areas of the sections appeared darker coloured than the rest (Additional file 1: Figure S1a, b). When sections of these darker coloured areas were examined by fluorescence microscopy, the lignin distribution in the tracheid walls indicated they were SCW. These darker coloured areas also appeared darker when the sections were examined in transmitted light (Additional file 1: Figure S1c, d). However, the boundary of the SCW could not be accurately determined by colour. Furthermore, MCWs could not be reliably distinguished from SCW or OW by colour.

### The three grades of CW have different tracheid wall structures

**Fig. 2:**
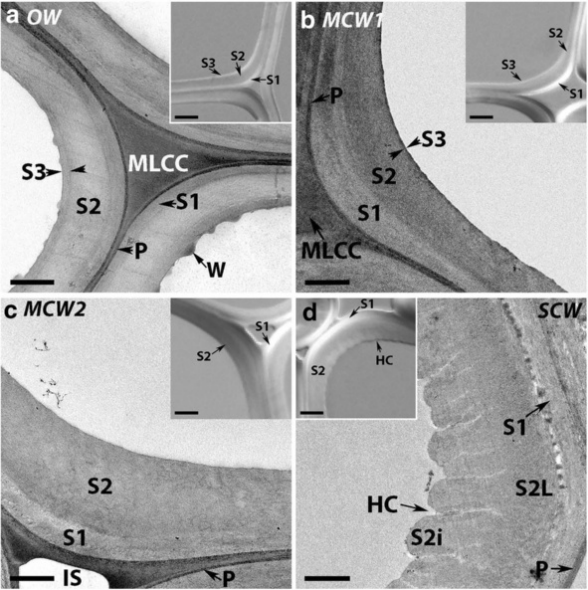
Micrographs of transverse sections of OW and three CWs showing tracheid wall structures. The main panels show transmission electron micrographs of OW (**a**), MCM1 (**b**), MCW2 (**c**) and SCW (**d**). In all wood types, the middle lamella is clearly differentiated from the primary wall (P) around the cells and at the cell corners (MLCC). All tracheid walls have a S1 and S2 layer, but an S3 layer is present in only OW (**a**) and MCW1 (**b**). Warts (W) are observed on the tracheid wall surface adjacent to the cell lumen in only OW (**a**). Helical cavities (HC) are present in the inner region of the S2 layer (S2i) in only SCW. Intercellular spaces (IS) are present between tracheids in only MCW 2 (**c**) and SCW (**d**). Micrographs obtained using a Leica confocal microscope. Scale bar: 1 μm. The insets show differential interference contrast micrographs. These particularly show the S1 and S3 layers in OW (**a**) and MCW1 (**b**) and helical cavities in SCW (**d**). All sections were from Tree 1. Scale bar: 5 μm

Examination of transverse sections of tracheid walls of all four wood types using transmission electron microscopy showed differences in wall structures (Fig. 2). Total tracheid wall thickness increased progressively in the order OW, MCW1, MCW2 and SCW. In all the wood types, the ML and primary wall could be differentiated and were densely stained. S1 and S2 secondary wall layers were also evident in all wood types. A well defined S3 layer was present in OW (Fig. 2a) and a very thin S3 layer could just be discerned in MCW1 (Fig. 2b), but no S3 layers were found in MCW2 or SCW (Fig. 2c, d). Helical cavities were present in the inner region of the S2 layer (S2i) only in SCW (Fig. 2d). Warts were observed on the tracheid wall surface adjacent to the cell lumen only in OW (Fig. 2a).

Differential interference micrographs (insets in Fig. 2) also showed the different layers in the tracheid secondary tracheid walls of the different wood types. These micrographs particularly showed the S1 layer in the tracheid walls in all the wood types, the S3 layer in OW and even in MCW 1, as well as the helical cavities in the S2i region of SCW.

### (1 → 4)-β-Galactans occur as a band in the S2L region of tracheid walls in all three grades of CW, with the band becoming wider with increasing severity

**Fig. 3:**
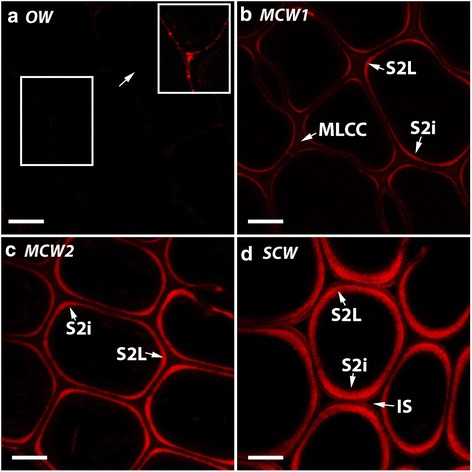
Immunofluorescence micrographs of transverse sections of tracheids of OW and three CWs labelled with LM5. OW (**a**) MCW1 (**b**), MCW2 (**c**) and SCW (**d**). There is extremely weak fluorescence of the tracheid walls in the tracheid walls of OW (**a**). Computer enhanced brightening of the selected region shows the CML, probably the primary walls, is the structure labelled (inset in (**a**) at the same scale). In MCW1, there is a thin band of fluorescence in the outer region of the S2 layer (S2L) which is brighter at the cell corners (**b**). In MCW2, this fluorescent band is wider and brighter than in MCW1, with the brightest part again at the cell corners (**c**). In SCW, this fluorescent band is even wider and brighter, but is of similar brightness all around the cell (**d**). All sections were from Tree 1. Scale bar: 10 μm

Immunofluorescence microscopy with the monoclonal antibody LM5, which specifically recognizes (1 → 4)-β-galactans, showed extremely weak labelling of the tracheid walls in OW (Fig. 3a). Computer enhanced brightening of the selected region of the image showed that the CML, probably the primary wall, was the structure that was labelled (see inset in Fig. 3a). In the MCW1 tracheid walls, there was a thin band of labelling corresponding to the outer region of the S2 layer, with the brightest labelling at the cell corners (Fig. 3b). In the MCW2 tracheid walls, the band was wider and brighter than in MCW1, with the brightest part again at the cell corners (Fig. 3c). The position of the band corresponds to the S2L layer. In the SCW tracheid walls, the band was even wider and brighter than in the MCW2 tracheid walls. Furthermore, in the SCW tracheid walls, the band was of similar brightness all around the cell (Fig. 3d). Thus, although only small amounts of (1 → 4)-β-galactan labelling were found in the tracheid walls in OW, in the CWs, more intense and greater areas of labelling were found in the outer layer of the S2 with increasing CW severity. No labelling was detected in micrographs from control experiments in which the primary antibody (LM5) was omitted.

**Fig. 4:**
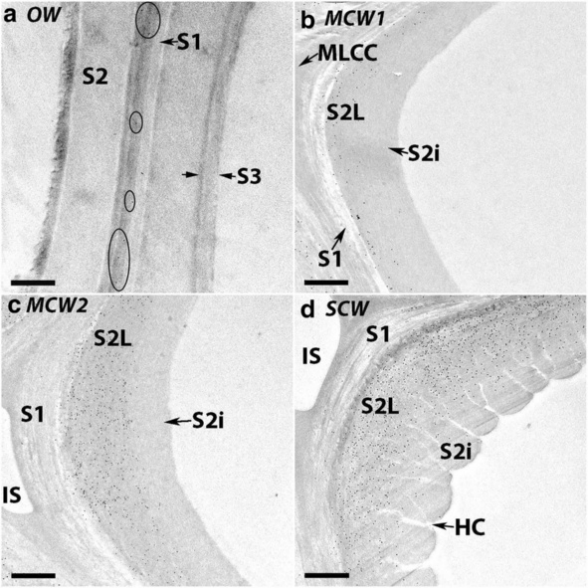
Immunogold micrographs of transverse sections of tracheids of OW and three CWs labelled with LM5. OW (**a**) MCW1 (**b**), MCW2 (**c**) and SCW (**d**). In the OW, only occasional particles (*circled*) are present over the CML, probably the primary wall of OW (**a**). In MCW1, particles are present as a band in the outer S2 layer (S2L) particularly at the cell corners, with smaller amounts in the S1 layer (**b**). The labelling density of the band increases with CW severity, particularly from MCW 1 to MCW 2 (**c**) in the S2L. The band of labelling in the SCW wall extends into the regions with helical cavities (HC) (**d**). All sections were from Tree 1. Scale bar: 1 μm

Immunogold microscopy with LM5 showed a similar pattern of labelling (Fig. 4). The OW tracheid walls showed occasional particles over only the CML region, probably over the primary wall (Fig. 4a circled), but all CW tracheids showed labelling as a band in the outer region of the S2 layer (S2L), with a smaller amount of labelling in the S1 layer (Fig. 4b-d). The band of labelling progressively increased in width from MCW1, MCW2 to SCW, with the labelling density increasing particularly from MCW1 (Fig. 4b) to MCW2 (Fig. 4c). The labelling in the SCW walls extended into the region with helical cavities (Fig. 4d). In MCW1 and MCW2 there was more labelling at the cell corners than around the cells (Fig. 4b, c). No labelling was found in micrographs from control experiments in which the primary antibody was omitted.

### (1 → 3)-β-Glucans occur as a band in the S2i region of tracheid walls in all three grades of CW, with the band becoming wider with increasing severity

**Fig. 5:**
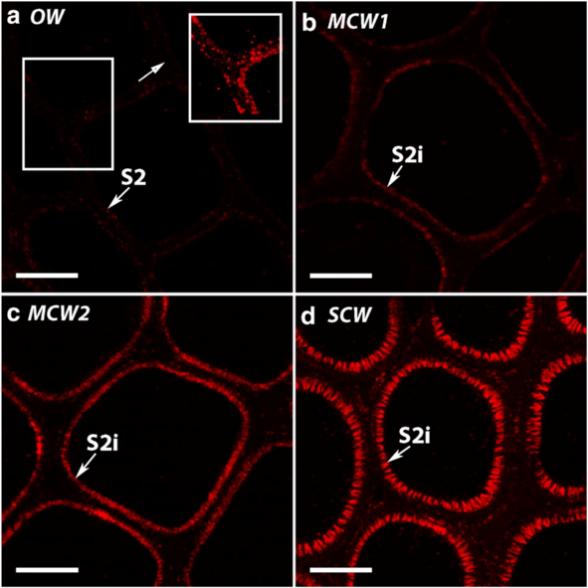
Immunofluorescence micrographs of transverse sections of OW and three CWs labelled with BS 400-2. OW (**a**) MCW1 (**b**), MCW2 (**c**) and SCW (**d**). In OW, there is very weak labelling of the tracheid walls (**a**). Computer enhanced brightening of the selected region (inset in **a** at the same scale) shows the S2 layer is the structure labelled. In MCW1, there is weak labelling of the inner region of the S2 layer (S2i) (**b**). This region is labelled brighter in MCW 2 (**c**). The brightest labelling is found in the same region in the SCW tracheid walls and is in the helical cavities. Sections were from Tree 1 and the micrographs obtained using a Leica confocal microscope. Scale bar: 10 μm

Immunofluorescence microscopy with the monoclonal antibody BS 400-2, which specifically recognizes (1 → 3)-β-glucans, showed very weak labelling of the tracheid walls in the OW (Fig. 5a). Computer enhanced brightening of the selected region of the image showed that the S2 tracheid wall layer was the structure labelled (see inset in Fig. 5a). The S2i region was weakly labelled in the MCW1 tracheid walls (Fig. 5b). This region was labelled brighter in the MCW2 tracheid walls (Fig. 5c), but the brightest labelling was in the same region of the SCW tracheid walls (Fig. 5d). In these SCW walls, the labelling was banded, corresponding to the helical cavities in this wall region. No labelling was found in micrographs from control experiments in which the primary antibody was omitted, or from control experiments using BS 400-2 that had been pre-incubated with laminarin.

**Fig. 6:**
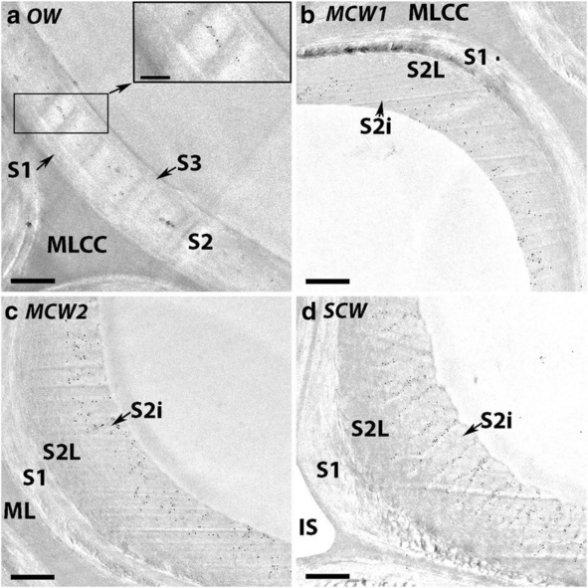
Immunogold micrographs of transverse sections of OW and three CWs labelled with BS 400-2. OW (**a**) MCW1 (**b**), MCW2 (**c**) and SCW (**d**). In OW tracheid walls, there are sporadic particles in the mid region of the S2 wall layer (see inset). In MCW1, there is denser labelling in the inner region of the S2 layer (S2i). Even greater density of labelling is found in the same wall region in MCW2. In SCW, there is abundant labelling of the S2i layer, which is mostly confined to within the helical cavities. Sections were from Tree 1. Scale bar: 1 μm (inset scale bar: 0.5 μm)

Immunogold microscopy with BS 400-2 showed a similar labelling pattern (Fig. 6). In the OW, occasional particles were found in the S2 tracheid wall layer. These were located in the mid region of this layer (inset Fig. 6a) rather than in the inner (S2i) or outer region (Fig. 6a). No label was present in the S1 or the S3 layers. Much more labelling was found in the MCW1 and this was present as a band in only the S2i region (Fig. 6b). Greater labelling density was found in the same wall region in the MCW2 tracheid walls (Fig. 6c). In the SCW, the labelling was again confined to the S2i region, and was mainly located in the helical cavities (Fig. 6d). No particles were found in micrographs from control experiments in which the primary antibody was omitted, or from control experiments using BS 400-2 that had been pre-incubated with laminarin.

**Fig. 7:**
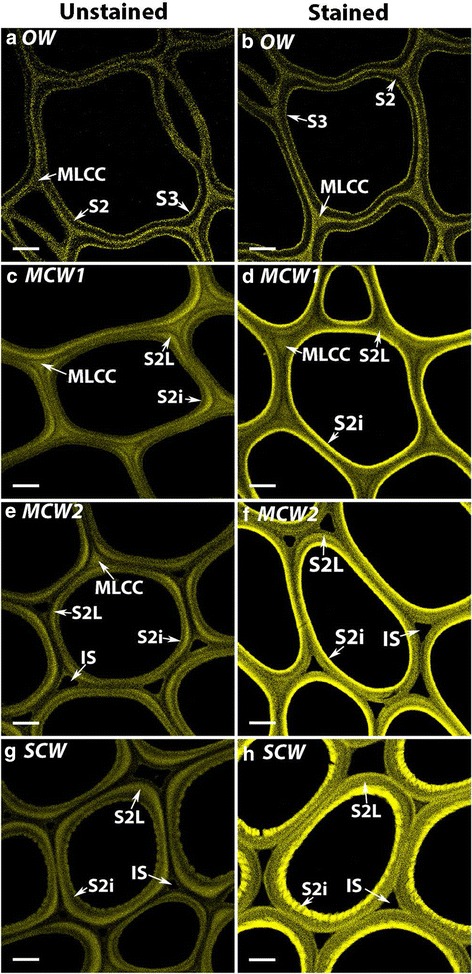
Fluorescence micrographs of transverse sections of OW and three CWs stained with aniline blue fluorochrome. OW unstained control (**a**) and stained (**b**); MCW1 unstained control (**c**) and stained (**d**); MCW2 unstained control (**e**) and stained (**f**); SCW unstained control (**g**) and stained (**h**). For the OW, there is no obvious difference between micrographs of the unstained, control section (**a**) and the stained section (**b**). For MCW1, MCW2 and SCW, the stained sections (**d**, **f**, **h**) show more fluorescence than the controls (**c**, **e**, **g**), with the most fluorescence being in the S2i region. Fluorescence intensity of this region was least in MCW1 (**d**) and most in SCW (**h**). Intercellular spaces (IS) are present only in MCW2 and SCW. Sections were from Tree 1 and the micrographs obtained using a Leica confocal microscope. Scale bar: 5 μm

The presence of (1 → 3)-β-glucans in the S2i region of the tracheid walls of all three grades of CW was also shown by staining sections with pure, synthetic aniline blue fluorochrome, which specifically binds to (1 → 3)-β-glucans (Fig. 7). Because lignin autofluoresces at the wavelengths used for this fluorochrome, fluorescence images were compared from sections of each wood type stained with the fluorochrome with unstained control sections. Lignin autofluorescence was also reduced by using the 458 nm laser line for excitation rather than the 488 nm line. For OW, there were no obvious differences between the micrographs of unstained and stained sections (Fig. 7a, b). However for the CWs, there was staining by the fluorochrome of the S2i region of the tracheid walls, with the staining intensity greatest for the SCW (Fig. 7g, h) and least for the MCW1 (Fig. 7c, d).

### (1 → 4)-β-Galactans labelling in whole-stem sections of tilted stems co-locates with CW

**Fig. 8:**
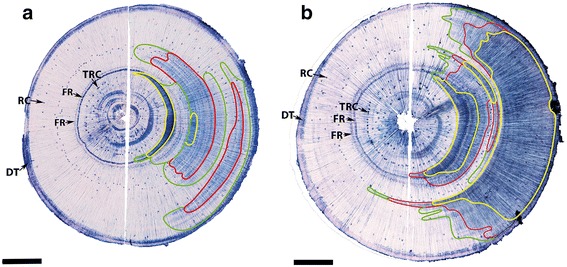
Immunolabelling of whole-stem transverse sections of tilted radiata pine saplings using LM5. Tree 3 (**a**) and Tree 1 (**b**). Blue coloration indicates the presence of (1 → 4)-β-galactans in cell walls. SCW labels dark blue (*outlined in yellow*), MCW 2 labelled mid blue (*outlined in red*) and MCW 1 labelled light blue (*outlined in green*). The grades of CWs were determined by the distribution of lignin in the tracheid walls using fluorescence microscopy. The cambium and adjacent differentiating tracheids (DT), normal resin canals (RC), traumatic resin canals (TRC), and two false growth rings (FR) were also labelled. Rays were also labelled and can just be seen as fine, light-blue, radial lines, particularly in (**b**). Scale bar: 5 mm

LM5 was also used in conjunction with a secondary antibody conjugated with an enzyme (alkaline phosphatase) to examine the distribution of (1 → 4)-β-galactans in whole sections of the tilted stems. The formation of an insoluble blue dye marked the locations of the (1 → 4)-β-galactans, which could be observed with the naked eye or low-power (stereo) microscope. On adjacent sections to ones used for immunolabelling, the locations of the four different wood types were determined based on lignin distributions in tracheid walls using fluorescence microscopy. Comparison of the distribution of blue coloration and its intensity among the wood types showed that OW gave no blue colour, MCW1 labelled light blue (outlined in green), MCW2 labelled mid blue (outlined in red) and SCW labelled dark blue (outlined in yellow) (Fig. 8). Labelled sections from Tree 3 (Fig. 8a), which was tilted at ~8° from the vertical, contained only small areas of SCW, but large areas of MCW1 and MCW2, whereas sections from Tree 1 (Fig. 8b), tilted at ~20°, contained large areas of SCW, but only small areas of MCW1 and MCW2.

In addition to CW, some other tissues were labelled. These included the cambium and adjacent differentiating tracheids before the formation of the secondary walls or the deposition of lignin; this occurred adjacent to both OW and CW. They also included resin canals, where the walls of the parenchyma cells surrounding the canals were labelled blue. Two types of resin canals were recognized in both the OW and CW: one type occurred singly and scattered, which we consider to be normal resin canals and the other, occurring in pairs in poorly defined rings, which we consider are traumatic resin canals (Fig. 8). To further investigate the occurrence of (1 → 4)-β-galactans in the walls of the parenchyma cells of resin canals, immunofluorescence microscopy (using a confocal laser scanning microscope) was carried out with LM5 on the OW side of the sections where the canals are present. This confirmed that (1 → 4)-β-galactans occurred sparsely in the thin walls of the epithelial cells surrounding the canals, but abundantly in the surrounding parenchyma cells of both normal and traumatic resin canals (Additional file 2: Figure S2b, d). However, labelling was not present in the walls of adjacent tracheids. Autofluorescence micrographs of the same areas obtained using the 488 nm laser line for excitation showed the distribution of lignin and other fluorescent materials; it showed the cell walls of the tracheids, the resin canal epithelial and parenchyma cells (Additional file 2: Figure S2a, c).

Ray cell walls were also labelled with LM5. In the immunolabelling of the whole-stem sections, these showed as light-blue radial lines throughout the sections, even in the dark blue regions. That these lines were due to the labelling of ray cell walls was confirmed using immunofluorescence microscopy (Additional file 2: Figure S2b, d, Additional file 3: Figure S3b). Labelling of the ray cell walls was much brighter than the walls of adjacent OW tracheids. However, the ray cell walls in SCW, labelled less brightly than the S2L layer of the walls of neighbouring tracheids (Additional file 3: Figure S3d). Autofluorescence micrographs of the same areas obtained using the 488 nm laser line, as indicated above, showed the walls of the tracheids adjacent to the rays (Additional file 3: Figure S3a, c).

Two concentric rings also labelled blue in the whole-stem sections (Fig. 8), both of which we consider to be false growth rings. The inner ring (labelled dark blue) was in the latewood zone of first year growth and the outer ring (labelled light blue) was in the earlywood zone at the beginning of the second year growth. Both rings apparently contained traumatic tissue, including incompletely developed tracheids with thin walls and incomplete lignification and showed evidence of collapse (Additional file 4: Figure S4a inner ring, c outer ring). In addition, the ray cells were expanded within these rings. Immunofluorescence microscopy with LM5 of both rings showed labelling of the walls of these abnormal tracheids and ray cells, which is consistent with the blue labelling of the rings in the whole-stem sections (Additional file 4: Figure S4b inner ring, d outer ring).

### The percentage of galactose in acid hydrolysates is higher in CWs and indicates CW severity

There were significant differences among the neutral-monosaccharide compositions of the non-cellulosic polysaccharides of the four wood types (Table 2). In particular, the percentages of galactose was lowest in the OWs hydrolysates (8.4–8.8 %) and highest in those of SCW (49.7–50.5 %), with intermediate percentages in MCW1 and MCW2, showing that the percentages of galactose indicate CW severity. Even in the milder of the two MCWs, MCW1, the percentages of galactose in the hydrolysates (29.4–31.6 %) were much higher than in those of the OW. The percentages of mannose, xylose and arabinose all decreased with wood type in the order OW, MCW1, MCW2, and SCW. Mannose had the highest percentage of all neutral monosaccharides in the OW (36.5–40.4 %), but the lowest percentage of the SCW (16.1–18.9 %). The percentage of glucose remained approximately similar among the wood types. There were some relatively small differences in the neutral monosaccharide percentages among the three different trees (*P*-value = 2.49 × 10^−6^). This was largely due to the percentage of glucose (Table 2). However, the situation was complicated by clear evidence that the differences between individual trees depends on the wood type (interaction *P*-value = 1.31 × 10^−5^). To display these differences, the plot of canonical variates 1 and 2 is shown in Additional file 5: Figure S5. This shows clearly the trend among the wood types (CV 1) and the differences between the individual trees (CV 2). The circles around each centroid are approximate confidence ellipses (95 %). The interaction effect that the differences between trees depend on the wood type is especially visible in MCW1, where the differences between the trees virtually vanish. Interestingly, in the other three wood types (OW, MCW2 and SCW) the two ramets from the same clone (Trees 1 and 2) are consistently different on CV2. This CV is largely associated with differences in the percentage of glucose. Table 3 shows the correlations between the neutral monosaccharide percentages and the canonical variate.

**Table 2:** Neutral monosaccharide composition (% w/w of all neutral monosaccharides) of the non-cellulosic polysaccharides in the four wood types from three trees

		Monosaccharides				
Tree	Wood type	Arabinose	Xylose	Galactose	Mannose	Glucose
Tree 1	OW	9.9 ± 0.6	30.3 ± 1.18	8.4 ± 1.4	36.5 ± 0.9	14.9 ± 1.0
(Tilted at ~20°)	MCW1	6.8 ± 0.5	20.9 ± 0.34	31.6 ± 0.7	27.7 ± 0.2	13.0 ± 1.3
	MCW2	5.7 ± 0.6	18.0 ± 0.86	40.8 ± 0.6	20.8 ± 1.0	14.8 ± 1.5
	SCW	4.9 ± 0.6	15.3 ± 0.90	49.7 ± 0.5	16.1 ± 1.4	14.0 ± 0.3
Tree 2	OW	11.0 ± 1.0	31.0 ± 1.1	8.8 ± 0.3	38.2 ± 0.6	11.0 ± 0.5
(Tilted at ~13°)	MCW1	6.3 ± 0.3	23.6 ± 0.2	30.4 ± 0.9	28.6 ± 0.7	11.1 ± 0.3
	MCW2	5.7 ± 0.5	17.8 ± 0.5	41.0 ± 0.9	20.7 ± 0.6	14.8 ± 1.4
	SCW	4.1 ± 0.2	14.2 ± 0.7	50.5 ± 1.1	18.9 ± 0.4	12.3 ± 0.8
Tree 3	OW	8.8 ± 0.4	30.1 ± 0.2	8.7 ± 0.7	40.4 ± 1.1	12.1 ± 0.1
(Tilted at ~8°)	MCW1	6.2 ± 0.8	23.4 ± 1.1	29.4 ± 0.2	30.3 ± 0.3	10.7 ± 0.3
	MCW2	5.3 ± 0.2	16.7 ± 0.9	41.1 ± 0.5	21.1 ± 1.0	15.9 ± 0.5
	SCW	3.5 ± 0.2	16.6 ± 0.3	49.9 ± 1.1	18.4 ± 0.2	11.6 ± 0.9

Means and standard errors of determination on three hydrolysates

**Table 3:** Structural coefficients (correlations) for the first two canonical variates (CV 1 and CV 2) of the differences between the neutral monosaccharide compositions of the non-cellulosic polysaccharides of the four wood types

	Monosaccharides				
CV	Arabinose	Xylose	Mannose	Galactose	Glucose
CV1	−0.962	−0.992	−0.984	0.999	0.243
CV2	0.201	−0.06	−0.17	−0.011	0.844

## Discussion

The present study showed that the two grades of MCWs we identified, based on the distribution of lignin in the tracheid walls, had tracheid wall structures and polysaccharide compositions intermediate between those in OW and SCW. A thin S3 wall layer similar to that found in our mildest grade, MCW1, has been reported in some samples of MCWs of the same species [19, 20]. Such a layer was also reported in the tracheid walls of very MCW in white spruce (*Picea glauca*). This finding came from a study, using ultraviolet-microscopy, of the transition between NW and SCW [16]. No S3 layer was found later in CW development and there have been no reports of it in the tracheid walls of SCW. However, another structural feature of tracheid walls, helical cavities in the S2i region, appears to be confined to SCW. The tracheid walls of neither of our two MCWs, MCW1 and MCW2, showed evidence of such cavities, and, as far as we are aware, there are no literature reports of these in the walls of MCW tracheids. Thus, in terms of tracheid wall structures in CWs, a S3 layer occurs in only the very mildest MCW, and helical cavities occur in the S2i region of only SCW.

In the present study, immunofluorescence and immunogold microscopy using the monoclonal antibody LM5, which is specific for (1 → 4)-β-galactans, showed these polysaccharides were located mostly as a band in the S2L layer of the tracheid walls of all three CW severities, although the band width increased with severity. Similar results have previously been reported for SCW in *P. radiata* using immunofluorescence microscopy [21–23] and immunogold microscopy [10, 22], in Sitka spruce (*Picea sitchensis*) also using both types of microscopy [10, 24] and in Norway spruce (*Picea abies*) using immunogold microscopy [10]. However, in none of these studies was MCW examined that had been defined using the distribution of lignin in the tracheid walls. Nevertheless, in the immunofluorescence microscopy study of Altaner et al. [24], some labelling was reported in the tracheid walls of what was described as “moderate CW” defined using only tracheid morphology. Interestingly, we found that at least some (1 → 4)-β-galactans were present in the tracheid wall S2L layer in even the mildest grade, MCW1, in which lignin was detected in the outer region of the S2L layer only at the cell corners. The presence of this polysaccharide is thus a characteristic feature of the S2L layer of tracheid walls in all grades of CW and not just SCW.

In contrast to the CWs, OW showed only weak labelling of the tracheid wall and this was at a different location, the compound middle lamella. This may represent labelling of (1 → 4)-β-galactan side chains of the pectic polysaccharide rhamnogalacturonan I (RG I), which is known to occur in the primary cell walls of coniferous gymnosperms [25]. RG I has been chemically characterized from the primary walls of cell suspension cultures of Douglas fir (*Pseudotsuga menziesii*) [26] and from cell walls in the differentiating xylem zone of Japanese cedar (*Cryptomeria japonica*) [27]. Both studies indicated that the RG I side chains contained much smaller proportions of (1 → 4)-β-galactans than of (1 → 5)-α-arabinans. Similar weak labelling of the primary wall with LM5 has been reported in tracheid walls of OW of radiata pine using immunofluorescence microscopy [23] and of radiata pine, Sitka spruce and Norway spruce using immunogold microscopy [10]. Consistent with this, only small amounts of (1 → 4)-β-galactans have been found in 6 M sodium hydroxide extracts of radiata pine OW compared with SCW [28]. These polysaccharides were also found in smaller amounts in OW than SCW in the same species using 2D NMR spectroscopy of cell-wall gels of finely milled wood [4]. The tracheid walls of OW therefore differed from those of CWs in only weakly labelling with LM5 and at a different location, the primary cell walls, where the labelling was probably due to (1 → 4)-β-galactans side chains on RG I.

Immunolabelling with LM5 of whole stem sections using an enzyme labelled secondary antibody showed the locations of CW tracheids and the different grades of CW present could be differentiated from one another. The labelled SCW also corresponded to the dark areas in sections viewed by reflected or transmitted light. The method would be more reliable than reflected or transmitted light methods which rely on SCW often having a darker colour than NW or OW [29–31]. The chemical basis of this coloration is unknown, and the colour intensity of SCW varies with species [2]. Transmitted light methods, in particular, have confused CWs with latewood [11] and with heartwood [30]. Although immunolabelling of whole stem sections is labour intensive, it is a promising method for the reliable detection and location of different grades of CWs in coniferous gymnosperms and could potentially be partially automated and used commercially. It could be particularly useful in detecting MCW, large areas of which were formed in the tree tilted at only ~8°. The angle of tilt is likely to be the major factor in determining the area of MCW formed relative to SCW. The sections from the tree tilted at ~20° contained much less MCW, but this tree was from a different clone and so it is possible that clonal difference may, at least partly, affect the proportions of MCW.

Immunolabelling with LM5 of whole stem sections also showed labelling of other features, including cambial cells and adjacent differentiating cells, producing both OW and CW, as well as rays, and resin ducts. However, this labelling could easily be distinguished from labelling of CW. Cambial and differentiating tracheids have primary walls, which would be expected to contain RG I with some (1 → 4)-β-galactans side chains. However, the labelling was brighter than would be expected when compared with the immunofluorescence and immunogold labelling of the primary walls of mature tracheids. It is possible that the high lignin concentrations in the primary walls of mature tracheids may partially mask the epitope resulting in weaker labelling. Further examination of the ray cells using immunofluorescence microscopy with LM5 showed labelling of their walls, which are non-lignified primary walls, again indicating the likely presence in these walls of (1 → 4)-β-galactan side chains on RG I. Similar immunofluorescence labelling with LM5 of ray cell walls has previously been reported in OW and SCW in Sitka spruce [24] and radiata pine [23]. Normal and traumatic resin canals were also labelled in whole stem sections. Both types of canals when examined by immunofluorescence microscopy with LM5, showed that the walls of the epithelial cells lining the canals labelled weakly, whereas the walls of the surrounding parenchyma cells labelled brightly. Similar labelling with LM5 of normal resin canals in radiata pine has previously been reported by Donaldson and Knox [23]. Traumatic resin canals occurring in pairs in poorly defined rings have been reported in many species of the family Pinaceae, including radiata pine, in response to a variety of stresses, including wounding and drought stress [32, 33]. The occurrence of these traumatic resin canals in the present study is likely to be in response to the tilting of the trees [34]. In the present study, the walls of the parenchyma and epithelial cells of these traumatic resin canals labelled in a similar way to those of normal resin canals. Thus, immunolabelling of whole stem sections with LM5 labelled cambial and ray cells, as well as resin ducts, with the labelling probably being due to (1 → 4)-β-galactans side chains of RG I in non-lignified primary walls.

Two false growth rings were also labelled by LM5 in the whole stem sections. Such rings have been described previously as occurring after a variety of stresses, including water stress [35–37] and frost [38]. Given the sapling trees in the present study were well watered, drought is not a likely cause of the false growth ring. However, frosts are known to occur in the area where the trees were grown, and so it is possible they are frost rings. LM5 labelling of such rings has not previously been reported, but abnormal tracheid development resulting in collapsed cells, with poorly developed lignification, has been reported in these rings, together with the occurrence in them of expanded rays due to the proliferation of parenchyma cells in the false growth rings. The collapsed tracheids lacked fully developed secondary walls suggesting that differentiation of the tracheid stopped prematurely [35, 37] and the irregular lignification may relate to a failure in the mechanism of lignification [38]. The RG I in the primary walls of the abnormal cells may have higher proportions of (1 → 4)-β-galactan side chains than in the equivalent walls of normal cells, resulting in greater LM 5 labelling. Interestingly, cold stress has been reported to cause an increase in the proportion of (1 → 4)-β-galactan side chains in RG I in pea (*Pisum sativum*), with these side chains possibly acting as a cold protectant [39, 40]. The LM5 labelling of the two false growth rings thus resulted from the labelling of primary walls in abnormal cells, possibly containing higher proportions of (1 → 4)-β-galactan side chains on RG I.

Immunofluorescence and immunogold microscopy with the monoclonal antibody BS 400-2 specifically detected (1 → 3)-β-glucans (callose or laricinan) in the S2i region of tracheid walls in all the CWs, with most labelling being detected in the SCW and the least in MCW1. The presence and location of (1 → 3)-β-glucans in the walls of SCWs tracheids has previously been reported using the same antibody [10, 41]. Using immunofluorescence microscopy (1 → 3)-β-glucans were shown to be located in or between helical cavities in the inner region of the S2 layer of the of the tracheid walls in SCW of Sitka spruce (*Picea sitchensis*) [41]. A later study using immunogold microscopy with the three species radiata pine, Sitka spruce, and Norway spruce (*Picea abies*) showed the (1 → 3)-β-glucans were located within these helical cavities in the S2 wall layer of tracheids in SCW [10]. Our immunogold microscopy studies also showed that in the SCW, the (1 → 3)-β-glucans were within the helical cavities. Interestingly in the two MCWs, (1 → 3)-β-glucans were also present in the S2i region despite there being no cavities. This has implications for the possible functions of (1 → 3)-β-glucans in these walls. They have been suggested as acting as permeability barriers and leak sealants [42, 43] and more recently as functioning to resist compressive stresses [44]. The finding of (1 → 3)-β-glucans within the cavities led to the suggestion that they may act as sealants for these cavities [10], but because in the MCWs the (1 → 3)-β-glucans occur in the absence of cavities, this suggests this is not their only role. Another likely role of (1 → 3)-β-glucans in CW tracheid walls is their ability to resist gravitropic stresses generated on tilting stems. The presence of (1 → 3)-β-glucans is thus a characteristic feature of the inner S2i layer of tracheid walls in all grades of CWs and not just SCW.

Before the availability of monoclonal antibodies to (1 → 3)-β-glucans, fluorescence microscopy after staining with decolorized aniline blue had been used to detect and locate these polysaccharides in the tracheid walls of SCW. This method was used to locate the polysaccharide in the S2i region of tracheid walls in *Pinus strobus* [45] and in 19 other species of gymnosperms [46]. The method relies on the presence in aniline blue preparations of a fluorochrome that is produced during the manufacture of the dye. The fluorochrome binds specifically to (1 → 3)-β-glucan [47, 48]. However, the decolorized aniline blue is used at a high pH which increases the intensity of lignin autofluorescence, making it difficult to locate (1 → 3)-β-glucans in lignified walls [49]. To reduce this problem, we used a pure, synthetic preparation of the fluorochrome in water [18], and, as with immunofluorescence microscopy with BS 400-2, we found (1 → 3)-β-glucans in the S2i region of tracheid walls in all CW grades. Thus, staining with the pure, synthetic fluorochrome in water is a fast, convenient method of detecting (1 → 3)-β-glucans in the tracheid walls of all CW severities.

The neutral monosaccharide compositions of the four wood types examined in the present study showed increasing proportions of galactose from OW, through MCW1 and MCW2 to the SCW. To determine these compositions, trifluoroacetic acid (TFA) was used under conditions that are known not to hydrolyse crystalline cellulose [50] and is well suited to study non-cellulosic polysaccharides. As TFA can easily be removed, it allowed very small samples to be used which were examined before hydrolysis by fluorescence microscopy to determine the lignin distribution in the tracheid walls, and hence the wood category could be confirmed. By using relative percentages of neutral monosaccharides in the hydrolysate rather than absolute yield of each monosaccharide on a dry weight basis, accurate weighing of samples was unnecessary. Even in hydrolysates of MCW1, where lignification of the tracheid S2L layer was apparent only at the cell corners, the percentage galactose was over three times greater than in OW hydrolysates. Intermediate amounts of galactose (on a g/100 g oven dry wood basis) were also reported in hydrolysates of MCW of radiata pine using the traditional two-stage sulphuric acid method, which also hydrolyses crystalline cellulose [17]. Although determining monosaccharide compositions cannot of course differentiate between different galactose-containing polysaccharides, labelling of the tracheid walls of MCW1 with LM5 is consistent with the majority of the galactose coming from (1 → 4)-β-galactans. Another potential source of galactose in the form of arabino-3,6-galactans have been shown not to be present in similar wood samples [4]. Lower proportions of mannose in hydrolysates of the radiata pine CWs in our study are consistent with decreases in the proportions of the most abundant non-cellulosic polysaccharides in OW, *O*-acetyl-galactoglucomannans, which contain galactose, glucose and mannose in the ratio 0.1:1.0:3.7 [51]. Lower proportions of arabinose and xylose in the same CW hydrolysates are consistent with decreases in the proportions of the second most abundant non-cellulosic polysaccharides in OW, arabino(4-*O*-methylglucurono)xylans, which contain 4-*O*-methylglucuronic acid, xylose and arabinose in the ratio of 1.0:5.8:1.1 [52]. However, given the glucose content of the *O*-acetyl-galactoglucomannans, it is interesting that the proportions of glucose remained similar in the hydrolysates of all four wood types. Higher proportions of (1 → 3)-β-glucans in the CWs may at least partly offset the decrease in glucose from the heteromannans. The neutral monosaccharide compositions of the four wood types showed higher proportions of galactose in the CW polysaccharides and are consistent with the greater labelling with LM5 of the CW tracheid walls than those of OW, and with the extent of labelling being related to CW severity.

The (1 → 4)-β-galactans in CW tracheid walls may, as suggested above for (1 → 3)-β-glucans, act to resist the gravitropic stresses generated on tilting stems. The (1 → 4)-β-galactans are co-located with lignin, and both may act together. Both (1 → 4)-β-galactans and (1 → 3)-β-glucans swell markedly on hydration and this may play a role in resisting these stresses in the tree and also result in the greater longitudinal shrinkage on drying of SCW than NW or OW [4, 5], which is of considerable commercial importance.

## Conclusions

Two grades of MCW, MCW1 and MCW2, as well as SCW and OW were identified in radiata pine and characterized. A S3 tracheid wall layer was present in only OW and the mildest MCW, MCW1, and helical cavities were present only in the inner region of the S2 tracheid wall layer in SCW. (1 → 4)-β-Galactans were a characteristic feature of the outer S2L tracheid wall layer of all grades of CW and not just SCW, with the proportions increasing with CW severity. However, the tracheid walls of OW contained only small proportions of these galactans, which were located in the primary walls. The neutral monosaccharide compositions of the non-cellulosic polysaccharides of the four wood types were consistent with these different proportions of (1 → 4)-β-galactans. Antibody labelling for (1 → 4)-β-galactans can also be used with whole stem sections to identify regions occupied by the four different wood types with the naked eye or low-power microscope. (1 → 3)-β-Glucans were a characteristic feature of the S2i region of tracheid walls in all grades of CWs and not just SCW, with the proportions increasing with CW severity. Thus, (1 → 3)-β-glucans and (1 → 4)-β-galactans were present in the tracheid walls of even the mildest grade of CW (MCW1). If these polysaccharides play a causal role in SCW shrinking longitudinally more than NW or OW, then it is likely that this will also be true for MCW, and be of considerable commercial importance.

## Methods

### Wood samples

Saplings of *Pinus radiata* (D. Don) (radiata pine) (Forest Genetics Ltd, Rotorua, New Zealand) were grown outside at Harewood, Christchurch, New Zealand. Seedlings were planted, in September 2011, in 100 litre bags of potting mix containing slow-release fertiliser and were irrigated regularly. Three saplings were used: clone 30 ramets 1 and 2, and clone 17, which are referred to as Trees 1, 2, and 3, respectively. They were grown upright for 6 months and then, in February 2012, tilted by staking at ~8–20° from the vertical to produce CW and OW; they were harvested in June 2013. The exact angle of tilting, measured at harvest, was for Tree 1 ~ 20°, Tree 2 ~ 13° and Tree 3 ~ 8°. A segment (~10 cm long) was sawn from each stem ~20 cm above the potting mix and used for all the experiments. Lengths (1 cm) were cut transversally from the segment using a band saw, and the surface smoothed using a sliding microtome (Model HN 40, Jung, Heidelberg, Germany), moistened with water and photographed in reflected light using a digital camera (Canon model EOS 40D) with a macro lens (EF 100 mm 1:28 usm ultrasonic motor) (Canon Corp., Tokyo, Japan). Transverse sections (1 mm thick) were also cut from the segment with a band saw, moistened with water, illuminated by transmitted light and photographed (as above).

### Identifying and obtaining samples of the four wood types

Lengths (1 cm) were cut from the stem segments with a band saw, softened by soaking in water at 4 °C for three days, and transverse sections (60 μm thick) cut using the sliding microtome. These sections were cut into two equal halves with one half being a darker colour (CW) than the other. Each half was then divided again giving four quadrants, each of which was then scanned by fluorescence microscopy (see below) to identify the locations of the four wood types based on the lignin distribution in the tracheid walls. Then discs were cut using a Harris Uni-Core™ micro-punch sampler (diameter 0.5 mm) (ProSciTech, Australia) from areas containing only one wood type and were again checked by fluorescence microscopy, with discs containing more than one wood type being rejected. These discs were used to determine the neutral monosaccharide compositions of the cell-wall polysaccharides (see below).

### Fixation, embedding and sectioning

Samples (~1 mm tangential width × ~ 1 mm radial width × ~10 mm long) were cut from the 1 cm stem lengths at locations where each wood type had been identified in the 60 μm thick sections (see above). These samples were fixed using 2 % (w/v) paraformaldehyde and 0.1 % (w/v) glutaraldehyde in 100 mM sodium 1,4-piperazinediethanesulfonic acid (PIPES) buffer (pH 7.2) for 2 h at room temperature under vacuum, and dehydrated in an aqueous ethanol series (30, 50, 70, 90, 95, and 100 % (twice)) for 15 min in each concentration. All samples were then infiltrated with a 1:2 (v/v) mixture of medium grade LR White resin (London Resin Co. Ltd, Basingstoke, UK) and ethanol at room temperature on a rotator for 1 h, then with a 2:1 (v/v) mixture of resin and ethanol for 1 h, and finally in pure resin for 18 h. The resin was polymerized for 24 h at 60 °C. Sections (100 and 500 nm thick) were cut with a diamond knife using an ultramicrotome (Model EM UC6: Leica, Vienna, Austria). Sections (500 nm thick) from each block were checked for wood type using fluorescence microscopy (see below). Any block containing more than one wood type was discarded. In addition samples containing the two false growth rings, and normal and traumatic resin canals were fixed and embedded in the same way.

### Indirect immunofluorescence microscopy

Indirect immunofluorescence microscopy was done with resin sections (500 nm thick) collected on poly-l-lysine coated slides (Biolab Scientific, Auckland) and dried at 55 °C for 30 min. The sections were incubated in phosphate buffered saline (PBS) (0.01 M sodium phosphate buffer, pH 7,4; 0.14 M NaCl) containing 5 % (w/v) milk powder (0.1 % fat; Alpine, Dairyworks Ltd, Christchurch, New Zealand) (MP-PBS) for 1 h to block nonspecific binding sites. After washing in PBS (5×), the sections were incubated with the monoclonal antibody LM5 (0.2 mL; 1:10 dilution) (PlantProbes, Leeds, UK) or the monoclonal antibody BS 400-2 that specifically recognizes (1 → 3)-β-glucans [53] (Biosupplies, Parkville, VIC, Australia) (0.2 mL; 1:10 dilution of the solution obtained by reconstituting the freeze-dried antibody according to the manufacturer’s instructions) in MP-PBS, and then with the secondary antibodies goat anti-rat IgG (H + L) conjugated to Alexa Fluor® 546 (used with LM5) and goat anti-mouse IgG (H + L) conjugated to Alexa Fluor® 546 (used with BS 400-2) (0.2 mL; 1:200 dilution). Both primary and secondary antibodies were incubated for 2 h, and after each incubation the sections were washed with PBS (5×). Sections were then washed with water, mounted in AF1 antifadent (Citifluor Ltd, London, UK) and examined by confocal laser scanning microscopy. Control experiments were done with the primary antibody omitted and where the BS 400–2 was pre-incubated with laminarin (100 μg/ml), a (1 → 3)-β-glucan, (Sigma-Aldrich, St. Louis, MO, USA) for 30 min [53].

### Indirect immunolabelling of (1 → 4)-β-galactans in whole-stem sections

Transverse sections (60 μm thick) of whole, debarked stems were treated with LM5 as described above, except incubations with MP-PBS and the antibodies were done at 30 °C and not at room temperature. Also the secondary antibody was goat anti-rat IgG (H + L chains) secondary antibody conjugated with alkaline phosphatase (0.2 mL; 1:100 dilution) (Invitrogen, New Zealand) in MP-PBS, and after the last PBS wash, the sections were treated for 15 min with substrate solution containing bromo-4-chloro-3-indolyl phosphate (BCIP), nitro-blue tetrazolium (NBT) and water (1:1:8 v/v) (BCIP/NBT substrate kit, Invitrogen). Alkaline phosphatase reacts with the substrate to produce an insoluble blue product. The sections were then washed in water (5×) before mounting in Aqua-Mount medium (Thermo Fisher, New Zealand). Control experiments were also done with the primary antibody omitted and the images recorded using a digital camera (see above).

### Light microscopy

Tracheid wall features were examined in transverse sections (60 μm thick) mounted in 75 % aqueous glycerol by differential interference optics using a Leica microscope (model DMR; Leica, Wetzlar, Germany). Similar sections mounted in the same way were used to examine lignin distribution in tracheid walls by its autofluorescence using this microscope fitted with an I3 filter block (excitation filter BP450-490 nm, chromatic beam splitter 510 nm, and emission filter LP515 nm) and using a confocal laser scanning microscope (model TCS SP2; Leica) with the 488 nm line of an Ar/Ar-Kr laser for excitation and emission >530 nm. The DMR microscope was also used to check the distribution of lignin autofluorescence in resin sections (500 nm thick).

Sections for immunofluorescence microscopy were examined using a confocal laser scanning microscope with the 561 nm line of a diode-pumped solid state laser used for excitation and emission > 590 nm. A Leica microscope (model TCS SP2) was used for the microscopy of all the tracheid walls and a Zeiss (Oberkochen, Germany) microscope (model LSM 710) was used for the microscopy of the other cell types.

Resin sections (500 nm thick) were also stained with an aqueous solution of the pure, synthetic aniline blue fluorochrome (0.033 mg/ml) (Biosupplies Australia Pty Ltd, Victoria, Australia) for 1 h at room temperature. After washing in water, the sections were examined using the Leica confocal laser scanning microscope with the 458 nm line of the Ar/Ar-Kr laser for excitation and emission > 493 nm. Unstained, control sections were also examined. The 458 line, rather than the 488 line, was used to reduce lignin autofluorescence.

### Indirect immunogold microscopy and transmission electron microscopy

Ultrathin sections (100 nm thick) of samples of resin-embedded wood (see above) were cut using an ultramicrotome and collected on 200 mesh square nickel grids (ProSciTech, Australia). Sections were incubated in 5 % MP-PBS for 1 h at room temperature to block nonspecific binding sites. After washing in PBS (2×), the sections were incubated with the monoclonal antibody LM5 or BS 400-2 (both at 1:10 dilution in 5 % MP-PBS) at 4 °C overnight. After washing in PBS (5×), the sections were incubated with the following secondary antibodies: goat anti-rat IgG (H + L) (with LM5) or goat anti-mouse IgG (H + L) (with BS 400-2) (both at 1:10 dilution in 5 % MP-PBS), both conjugated to 15 nm diameter colloidal gold particles (Electron Microscopy Sciences, Hatfield, PA, USA) at room temperature for 2 h. Then all the sections were washed in PBS (5×) followed by water (2 ×). Control experiments were done in which the primary antibody was omitted and in which BS400-2 was pre-incubated with laminarin (100 μg/ml) for 30 min [53].

For studies not involving immunolabelling, freshly cut sections were treated with 2 % aqueous uranyl acetate for 20 min, washed with water (5×), stained with a solution of lead citrate for 2 min, washed with water (5×), and dried.

All sections were examined with a transmission electron microscope (Model CM12, Philips, Eindhoven, The Netherlands) operated at 80 kv.

### Determining the neutral monosaccharide compositions of the cell-wall polysaccharides

Neutral monosaccharide compositions of the non-cellulosic polysaccharides in the walls of the four wood types were determined using hydrolysates of small discs (0.5 mm diameter each containing ~610 tracheids) that had been checked by fluorescence microscopy to ensure that all tracheids walls had the appropriate lignin distributions (Additional file 6: Figure S6). A total of three discs for each wood type were cut from each section. Each disc was transferred to a separate glass vial, dried over silica gel for 16 h, and hydrolysed with 2 M trifluoroacetic acid (TFA) (0.1 mL, 121 °C, 1 h) in a sealed tube under argon [28, 54, 55]. After cooling, the 2 M TFA was evaporated in a stream of air, the residues dissolved in water (0.2 mL) and filtered using a PTFE filter (pore size 0.2 μm; WhatmanTM, Maidstone, Kent, UK). The neutral monosaccharides were separated and quantified using high-performance anion-exchange chromatography with pulsed amperometric detection (HPAEC-PAD) on a Dionex BioLC system (Dionex, Sunnyvale, CA, USA) fitted with an ED 50 electrochemical detector and a GP 50 gradient pump. A CarboPac PA 20 guard column (3 × 30 mm) and a CarboPac PA20 analytical column (3 × 150 mm) were used. Column temperature was kept at 25 °C by a TCC-100 thermostatted column compartment. Neutral monosaccharides were separated using isocratic elution (1 mM NaOH for 30 min). The column was washed for 5 min with 200 mM NaOH before equilibration for 10 min with 1 mM NaOH. The injection volume was 20 μL, and the flow rate was 0.4 mL min^−1^. The order of elution of monosaccharides was confirmed by running solutions of individual reference monosaccharides. Before the hydrolysate runs, a water blank was run following by a reference solution containing 0.01 mg mL^−1^ of each of l-arabinose, d-galactose, d-glucose, d-xylose and d-mannose, which was used to determine the relative responses of equal weighs of each monosaccharide. Mean neutral monosaccharide compositions were calculated from the compositions of the three discs of each wood type.

The neutral monosaccharide compositions were examined statistically. Since the data are multivariate (five monosaccharides), a 2-way factorial multivariate analysis of variance (MANOVA) was first done to search for differences between the wood types and individual trees. To display the differences between the centroids we used canonical discriminant analysis to plot the centroids in two dimensions with a minimum loss of information [56]. All statistical analysis was performed in R (version 3.0.1) [57].

## Additional files

Additional file 1:
**Figure S1.** Photographs of whole-stem transverse sections of tilted radiata pine saplings. Photographed in reflected light Tree 3 (a) and Tree 1 (b) and in transmitted light Tree 3 (c) and Tree 1 (d). Tree 1 was tilted at ~20° to the vertical and Tree 3 at ~8°. The darker coloured areas (arrows) contained SCW, determined by the distribution of lignin in the tracheid walls using fluorescence microscopy. Scale bars: 5 mm. (PDF 192 kb) Additional file 2:
**Figure S2.** Fluorescence and immunofluorescence micrographs of transverse resin sections of resin canals in OW labelled with LM5. Fluorescence micrographs of a normal resin canal (a) and a pair of traumatic resin canals (c) and immunofluorescence micrographs the same normal resin canal (b) and pair of traumatic resin canals (d). The fluorescence micrographs shows the distribution of lignin and other autofluorescent materials. The epithelial and parenchyma walls of both normal and traumatic resin canals and the tracheid walls show autofluorescence. The thin-walled epithelial cells (E) in both normal and traumatic resin canals are sparsely labelled with LM5. The walls of the parenchyma cells (P) in both types of canals are brightly labelled. The ray (R) cell walls are also brightly labelled. Sections were from Tree 1 and the micrographs obtained using a Zeiss confocal microscope. Scale bar: 20 μm. (PDF 312 kb) Additional file 3:
**Figure S3.** Fluorescence and immunofluorescence micrographs of transverse resin sections of rays in OW and SCW labelled with the monoclonal antibody LM5. Fluorescence micrographs of ray cells and adjacent tracheids of OW (a) and SCW (c), showing the distribution of lignin and other autofluorescent materials. The walls of the tracheids (T) are clearly seen and there is autofluorescent material in some of the ray cells (R). Immunofluorescence micrograph of OW (b) shows labelling of the ray cell walls, and of SCW (d) shows strong labelling of the S2L layer of the tracheid walls and weaker labelling of the ray cell walls. Sections were from Tree 1 and the micrographs obtained using a Zeiss confocal microscope. Scale bar: 50 μm. (PDF 168 kb) Additional file 4:
**Figure S4.** Fluorescence and immunofluorescence micrographs of transverse resin sections of two false growth rings labelled with the monoclonal antibody LM5. Fluorescence micrographs of the inner false growth ring (a) and the outer false growth ring (c), showing the distribution of lignin and other autofluorescent materials. Both false growth rings contain abnormal tissues, including thin-walled incompletely lignified tracheids (ILT), collapsed tracheids (CT), and expanded ray (ER) cells. Immunofluorescence micrographs of the inner false growth ring (b) and the outer false growth ring (d) show strong labelling of these abnormal tissues. Sections were from Tree 1 and the micrographs obtained using a Zeiss confocal microscope. Scale bar: 50 μm. (PDF 392 kb) Additional file 5:
**Figure S5.** Canonical discriminant analysis plot. The four wood types from three trees defined by the first two canonical variates (CV 1 and CV2) obtained from canonical discriminant analysis conducted on all neutral monosaccharide variables combined. The centroids for each wood type of each tree with approximate 95 % confidence regions are separated based on differences in the monosaccharide compositions between wood types, and not trees. The percentage in the axis labels refers to the proportion of the total between-centroid variance summarised by that canonical variate. (PDF 87 kb) Additional file 6:
**Figure S6.** Fluorescence micrograph of a 0.5 mm diameter disc cut from MCW1 using a micro-punch sampler. The micrograph shows the disc containing ~ 610 tracheids, with autofluorescent walls. The disc was from Tree 1 and the micrographs obtained using a DMR microscope. Scale bar: 100 μm. (PDF 80 kb)

## Abbreviations

CML: Compound middle lamella
CW: Compression wood
MCW: Mild compression wood
MCW1: Mild compression wood type 1
MCW2: Mild compression wood type 2
ML: Middle lamella
MLCC: Middle lamella at cell corners
NW: Normal wood
OW: Opposite wood
RG I: Rhamnogalacturonan I
S2i: Inner region of S2 layer
S2L: Outer region of S2 layer
SCW: Severe compression wood
