# Supplementary figures and images for: Tracheid cell-wall structures and locations of (1 → 4)-β-d-galactans and (1 → 3)-β-d-glucans in compression woods of radiata pine (Pinus radiata D. Don)
Source: BMC Plant Biol. 2016 Sep 7;16(1):194. doi: 10.1186/s12870-016-0884-3 (PMC5015220; doi:10.1186/s12870-016-0884-3)

**a**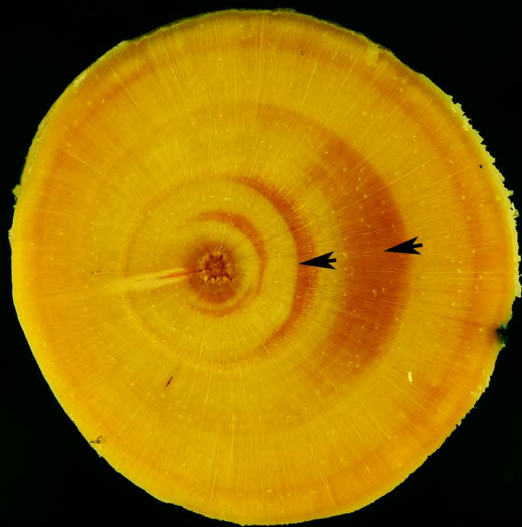**b**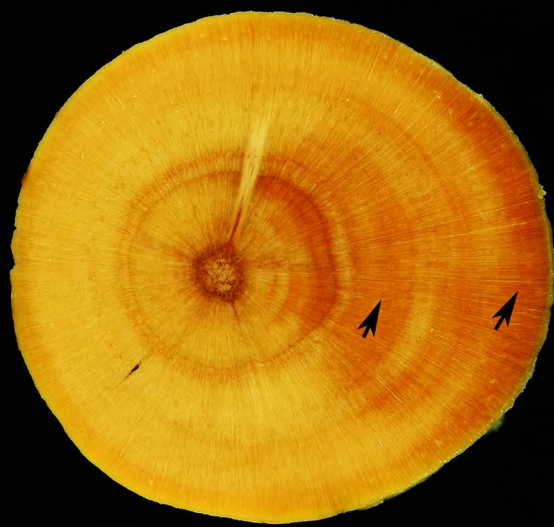**c**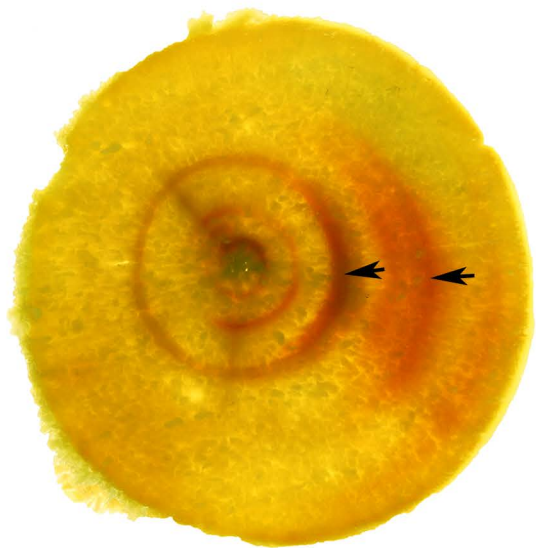**d**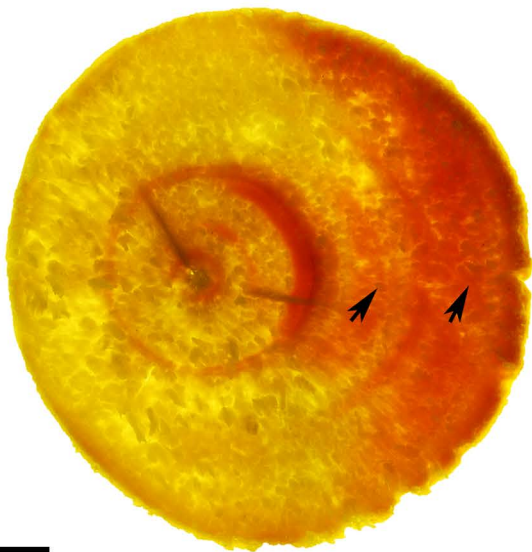

Supplement: Additional file 1: — Figure S1. Photographs of whole-stem transverse sections of tilted radiata pine saplings. Photographed in reflected light Tree 3 (a) and Tree 1 (b) and in transmitted light Tree 3 (c) and Tree 1 (d). Tree 1 was tilted at ~20° to the vertical and Tree 3 at ~8°. The darker coloured areas (arrows) contained SCW, determined by the distribution of lignin in the tracheid walls using fluorescence microscopy. Scale bars: 5 mm. (PDF 192 kb) [file 12870_2016_884_MOESM1_ESM.pdf]

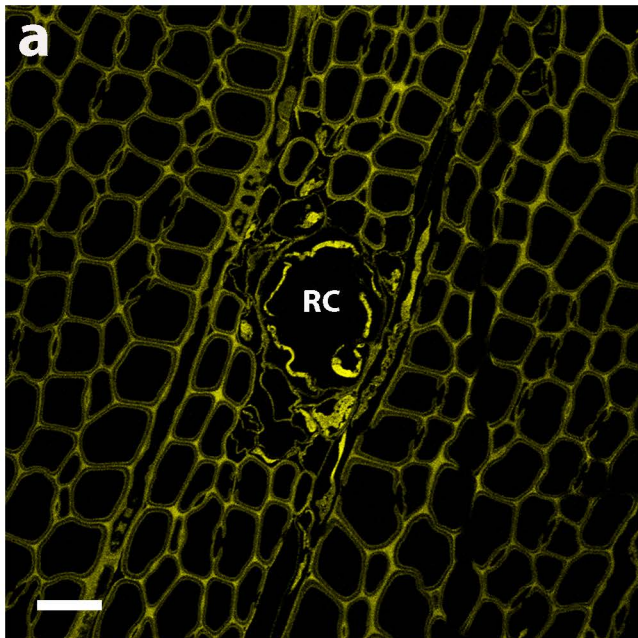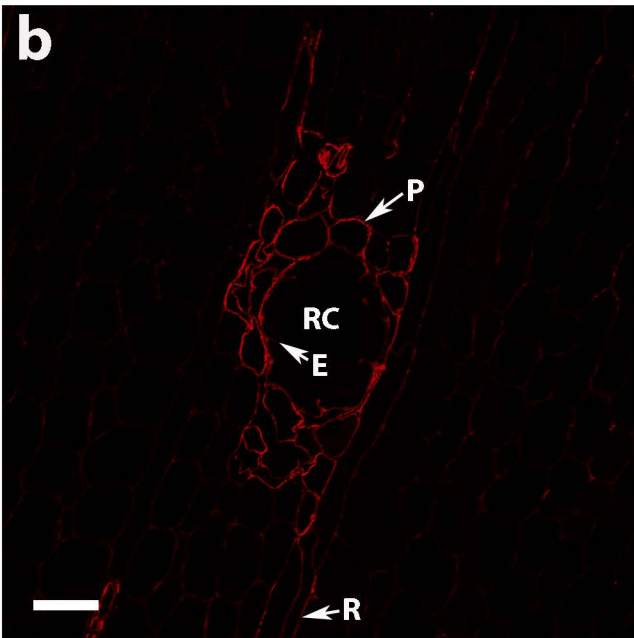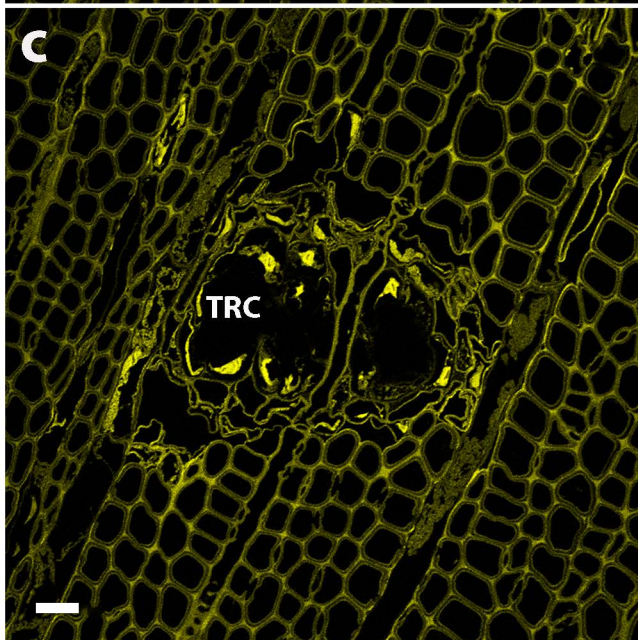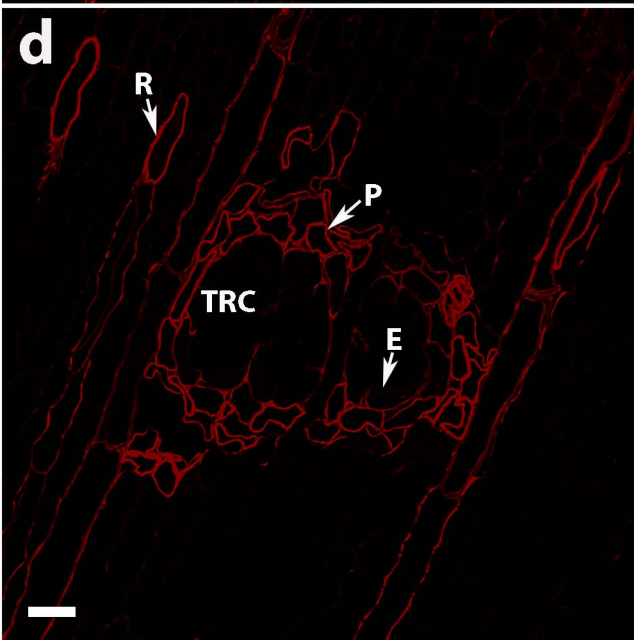

Supplement: Additional file 2: — Figure S2. Fluorescence and immunofluorescence micrographs of transverse resin sections of resin canals in OW labelled with LM5. Fluorescence micrographs of a normal resin canal (a) and a pair of traumatic resin canals (c) and immunofluorescence micrographs the same normal resin canal (b) and pair of traumatic resin canals (d). The fluorescence micrographs shows the distribution of lignin and other autofluorescent materials. The epithelial and parenchyma walls of both normal and traumatic resin canals and the tracheid walls show autofluorescence. The thin-walled epithelial cells (E) in both normal and traumatic resin canals are sparsely labelled with LM5. The walls of the parenchyma cells (P) in both types of canals are brightly labelled. The ray (R) cell walls are also brightly labelled. Sections were from Tree 1 and the micrographs obtained using a Zeiss confocal microscope. Scale bar: 20 μm. (PDF 312 kb) [file 12870_2016_884_MOESM2_ESM.pdf]

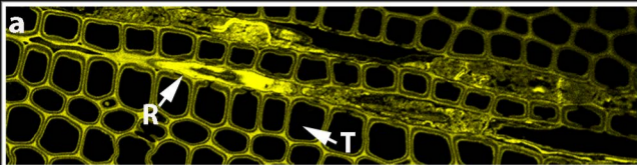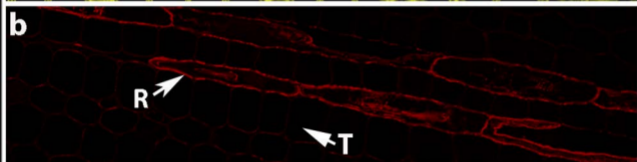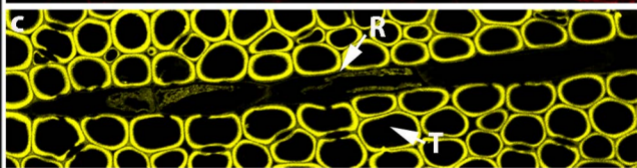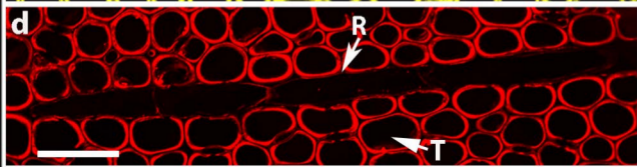

Supplement: Additional file 3: — Figure S3. Fluorescence and immunofluorescence micrographs of transverse resin sections of rays in OW and SCW labelled with the monoclonal antibody LM5. Fluorescence micrographs of ray cells and adjacent tracheids of OW (a) and SCW (c), showing the distribution of lignin and other autofluorescent materials. The walls of the tracheids (T) are clearly seen and there is autofluorescent material in some of the ray cells (R). Immunofluorescence micrograph of OW (b) shows labelling of the ray cell walls, and of SCW (d) shows strong labelling of the S2L layer of the tracheid walls and weaker labelling of the ray cell walls. Sections were from Tree 1 and the micrographs obtained using a Zeiss confocal microscope. Scale bar: 50 μm. (PDF 168 kb) [file 12870_2016_884_MOESM3_ESM.pdf]

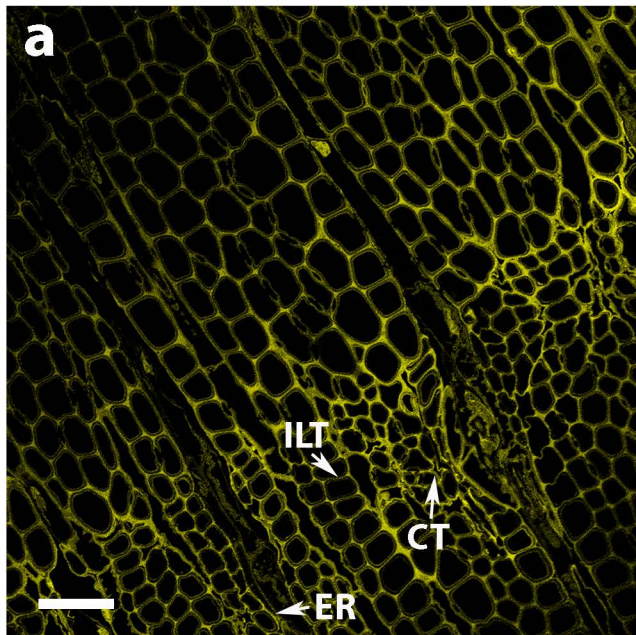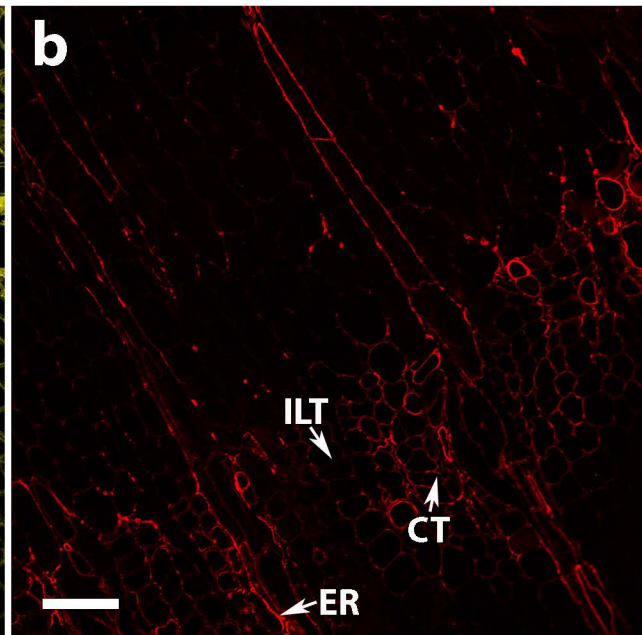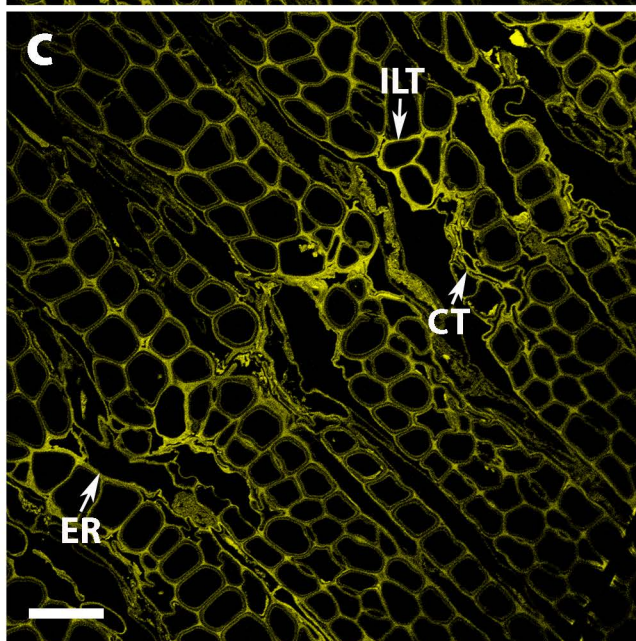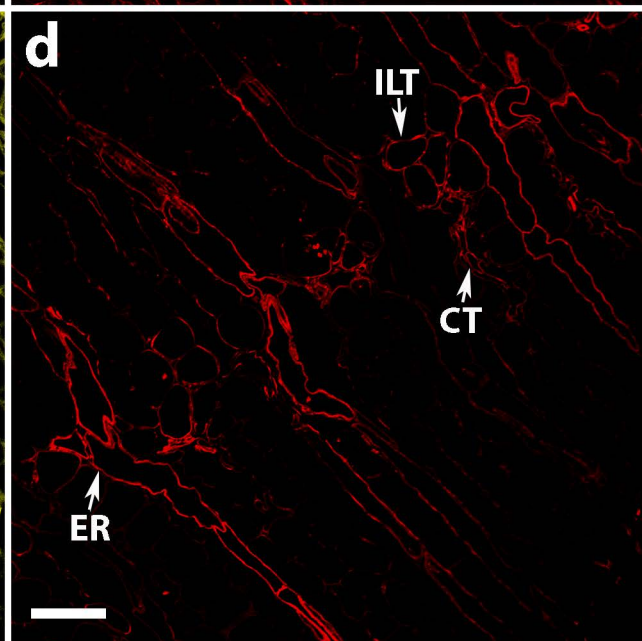

Supplement: Additional file 4: — Figure S4. Fluorescence and immunofluorescence micrographs of transverse resin sections of two false growth rings labelled with the monoclonal antibody LM5. Fluorescence micrographs of the inner false growth ring (a) and the outer false growth ring (c), showing the distribution of lignin and other autofluorescent materials. Both false growth rings contain abnormal tissues, including thin-walled incompletely lignified tracheids (ILT), collapsed tracheids (CT), and expanded ray (ER) cells. Immunofluorescence micrographs of the inner false growth ring (b) and the outer false growth ring (d) show strong labelling of these abnormal tissues. Sections were from Tree 1 and the micrographs obtained using a Zeiss confocal microscope. Scale bar: 50 μm. (PDF 392 kb) [file 12870_2016_884_MOESM4_ESM.pdf]

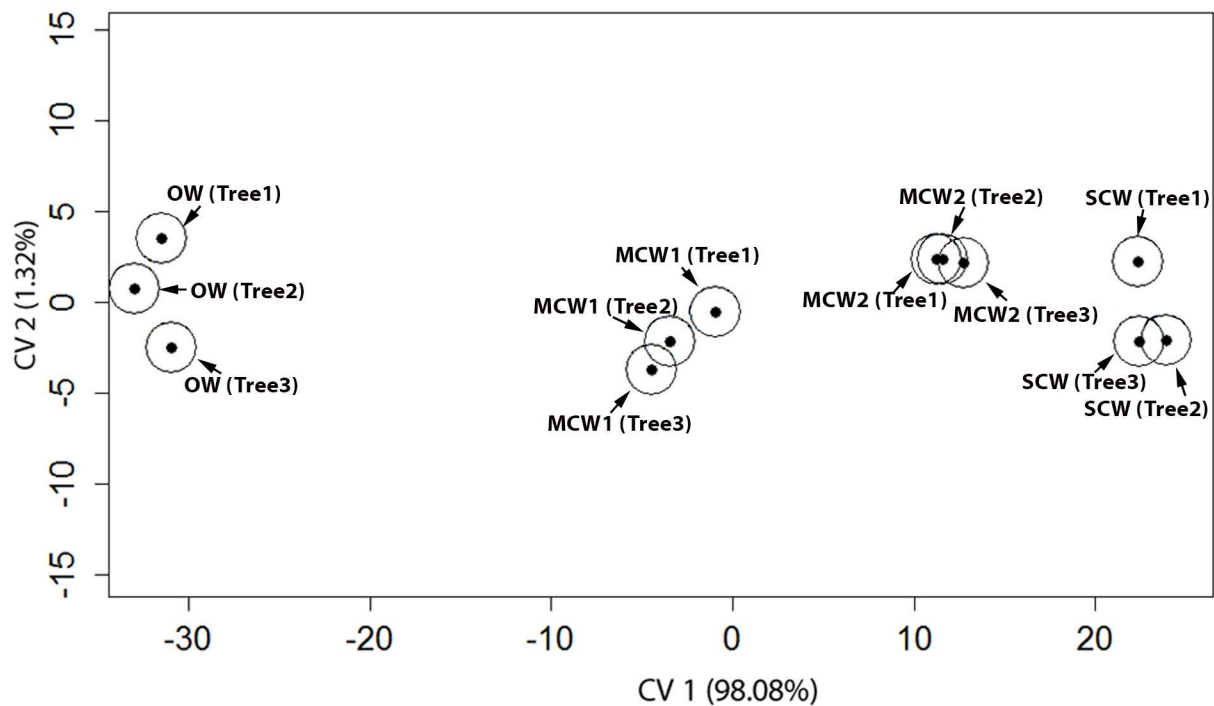

Supplement: Additional file 5: — Figure S5. Canonical discriminant analysis plot. The four wood types from three trees defined by the first two canonical variates (CV 1 and CV2) obtained from canonical discriminant analysis conducted on all neutral monosaccharide variables combined. The centroids for each wood type of each tree with approximate 95 % confidence regions are separated based on differences in the monosaccharide compositions between wood types, and not trees. The percentage in the axis labels refers to the proportion of the total between-centroid variance summarised by that canonical variate. (PDF 87 kb) [file 12870_2016_884_MOESM5_ESM.pdf]

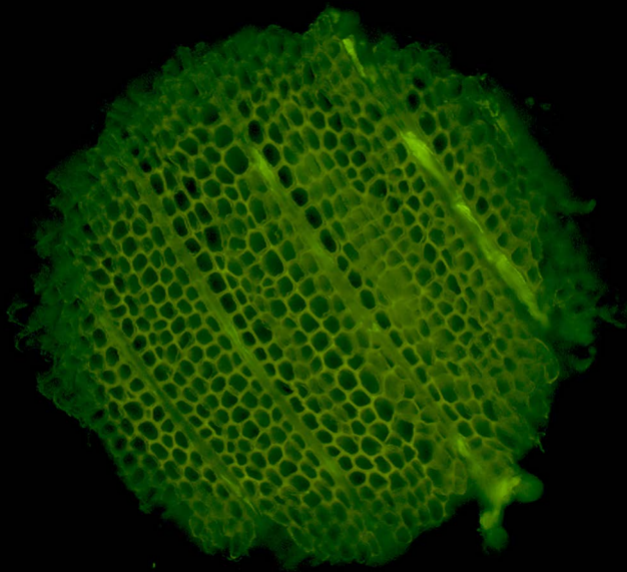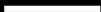

Supplement: Additional file 6: — Figure S6. Fluorescence micrograph of a 0.5 mm diameter disc cut from MCW1 using a micro-punch sampler. The micrograph shows the disc containing ~ 610 tracheids, with autofluorescent walls. The disc was from Tree 1 and the micrographs obtained using a DMR microscope. Scale bar: 100 μm. (PDF 80 kb) [file 12870_2016_884_MOESM6_ESM.pdf]
